# Therapeutic induction of Bcl2‐associated athanogene 3‐mediated autophagy in idiopathic pulmonary fibrosis

**DOI:** 10.1002/ctm2.935

**Published:** 2022-07-14

**Authors:** Shashipavan Chillappagari, Julian Schwarz, Vidyasagar Kesireddy, Jessica Knoell, Martina Korfei, Konrad Hoetzenecker, M. Lienhard Schmitz, Christian Behl, Saverio Bellusci, Andreas Guenther, Poornima Mahavadi

**Affiliations:** ^1^ Department of Internal Medicine Justus‐Liebig University (JLU) Giessen Giessen Hessen Germany; ^2^ Universities of Giessen and Marburg Lung Center (UGMLC) Member of the German Centre for Lung Research (DZL) Giessen Hessen Germany; ^3^ Department of Biochemistry Faculty of Medicine JLU Giessen Giessen Hessen Germany; ^4^ Department of Thoracic Surgery Vienna General Hospital Vienna Austria; ^5^ European IPF Network and European IPF Registry Giessen Germany; ^6^ Member of the Cardio‐Pulmonary Institute (CPI) JLU Giessen Giessen Germany; ^7^ Institute of Pathobiochemistry, The Autophagy Lab University Medical Center Johannes Gutenberg University Mainz Germany; ^8^ Lung Clinic Agaplesion Evangelisches Krankenhaus Mittelhessen Giessen Germany

**Keywords:** 5‐azacytidine, autophagy, BAG3, cantharidin, fibroblasts, filamin C, idiopathic pulmonary fibrosis, pirfenidone

## Abstract

**Background:**

Exaggerated fibroblast proliferation is a well‐known feature in idiopathic pulmonary fibrosis (IPF) which may be – in part – due to insufficient autophagy, a lysosome dependent cellular surveillance pathway. Bcl2‐associated athanogene 3 (BAG3) is a pivotal co‐chaperone of the autophagy pathway. Here, we studied whether therapeutic modulation of BAG3‐mediated autophagy can rescue insufficient autophagy and impact IPF fibroblast proliferation.

**Methods:**

Primary interstitial fibroblasts or precision cut lung slices (PCLS) of IPF lungs were treated with (1) the antifibrotic drug pirfenidone (Pirf), (2) the demethylating agent 5‐azacytidine (Aza), (3) the BAG3 modulator cantharidin (Ctd). Autophagy flux was measured following pretreatment with the autophagy inhibitors or by GFP‐RFP‐LC3B transfection followed by drug treatments. Proliferation was measured by 5‐bromo‐2′‐deoxyuridine assay. BAG3, filamin C (FLNC), proliferating‐cell‐nuclear‐antigen (PCNA), collagen1A1 (COL1A1) and autophagy proteins were assessed by immunoblotting or immunofluorescence. Loss of function experiments were performed by siRNA mediated knockdown of *BAG3*.

**Results:**

In comparison with healthy donors, increased BAG3 protein was observed in IPF lung homogenates and IPF fibroblasts. In addition, the substrate of BAG3‐mediated autophagy, FLNC, was increased in IPF fibroblasts, implying insufficient activation of BAG3‐dependent autophagy. Therapeutic modulation of this pathway using Aza and Ctd alone or in combination with the IPF therapy drug Pirf rescued the insufficient BAG3‐mediated autophagy and decreased fibroblast proliferation. Such effects were observed upon therapeutic modulation of BAG3 but not upon knock down of *BAG3* per se in IPF fibroblasts. Similarly, PCLS of IPF patients showed a significant decrease in collagen deposition in response to these drugs, either alone or in a more potent form in combination with Pirf.

**Conclusions:**

Our study reveals that repurposing drugs that modulate autophagy regulating proteins render therapeutic benefits in IPF. Fine tuning of this pathway may hence signify a promising therapeutic strategy to ameliorate antifibrotic properties and augment the efficacy of current IPF therapy.

## Graphical abstract

IPF‐fibroblasts are refractory to BAG3‐mediated autophagy. Knock down of *BAG3* per se in IPF fibroblasts does not ameliorate BAG3‐mediated autophagy. Therapeutic intervention (pirfenidone+5‐azacytidine or pirfenidone+cantharidin) sensitizes IPF fibroblasts to BAG3‐mediated autophagy. Drug‐induced sensitization of BAG3‐mediated autophagy in precision cut lung slices of IPF patients alleviates fibroblast proliferation and collagen deposition.

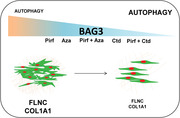

## 1 BACKGROUND

B‐cell lymphoma 2 (Bcl‐2) associated athanogenes (BAGs) belong to the BAG family of co‐chaperones that regulate diverse cellular processes. BAG family contains about six proteins (BAG1‐6), which contain at least one copy of their characteristic ‘BAG’ domain at the C‐terminus, allowing them to interact with the ATPase domain of the eukaryotic chaperone 70‐kilodalton heat shock protein (Hsp70).^1^, ^2^ BAG proteins interact with client proteins through their N‐terminus to target them to specific cellular locations.^3^ Of all the BAG proteins, BAG3 is the only protein reported to be involved in critical cell fate decisions by acting as an important co‐chaperone in the selective degradation of aggregated proteins via autophagy, a lysosome‐dependent quality control mechanism.^4^ This BAG3‐dependent selective autophagy that was initially found in Drosophila.^5^, ^6^ Here, BAG3 binds to the HSPA8‐associated ubiquitin ligase STUB1/CHIP and its partner UBE2D (ubiquitin‐conjugating enzyme E2D) and helps to ubiquitinate chaperone‐bound filamin C (FLNC). This in turn results in its degradation by autophagy via the recruitment of the selective autophagy substrate protein, sequestosome1 (p62/SQSTM1) in striated skeletal muscles.^5^ BAG3−/− mice develop fulminant myopathy characterized by noninflammatory myofibrillar degeneration.^7^, ^8^

Given the role of the BAG3‐mediated autophagy under pathological conditions,^9^, ^10^ it was interesting to study this autophagic pathway in idiopathic pulmonary fibrosis (IPF). IPF belongs to the group of idiopathic interstitial pneumonias (IIPs) according to the classification of interstitial lung diseases (ILDs) or diffuse parenchymal lung diseases (DPLDs).^11^ As the name suggests, IPF has an unknown aetiology and is the most important form of IIPs. It has a remarkable age‐related onset and represents one of the most aggressive forms of organ fibrosis, with a survival time of only a few years after diagnosis.^12^ Two drugs, pirfenidone (Pirf) and nintedanib, are approved for IPF therapy that slow down progression but do not reverse the disease process. Therefore, lung transplantation remains the only treatment option for late stage patients. IPF is characterized by a progressive decline in lung function and is associated with the histological and appearance of a usual interstitial pneumonia (UIP) pattern. Chronic epithelial injury, disturbed epithelial‐mesenchymal cross‐talk, activation of fibroblasts, excessive collagen deposition and disruption of the delicate alveolar architecture altogether result in progressive dyspnoea, decline in lung function and, ultimately, death.^13^, ^14^ In this context, the highly proliferative and apoptosis‐resistant behaviour of IPF fibroblasts contributes to the excessive and self‐perpetuating extracellular matrix deposition. In the past decade, lysosomal stress and a defective autophagy have been explicitly reported in several cell types of IPF patient lungs as well as in animal models of lung fibrosis.^15^, ^16^, ^17^, ^18^

Here, we studied the role and function of BAG3‐dependent autophagy in IPF fibroblasts and found that therapeutic induction of this pathway by different drugs reduced collagen deposition and fibroblast proliferation.

## 2 METHODS

### 2.1 Human lungs

Explanted lungs were obtained from sporadic IPF patients or nondiseased control subjects/healthy donors/organ donors (HD) from the Department of Thoracic Surgery, Vienna. Patient information and patient samples used for different experiments are tabulated in Table 1. All IPF diagnoses were made according to the American Thoracic Society/European Respiratory Society consensus criteria.^19^ The study protocol was approved by the Ethics Committee of the Justus‐Liebig‐University School of Medicine (111/08 and 58/15). The data and all patient‐related biomaterials used in this study were provided by the UGMLC Giessen Biobank and the European IPF Registry/Biobank.

**TABLE 1:** Patient information

Patient number	Age	Gender	Total lung homogenates	Primary fibroblasts	PCLS
IPF#1	52	M	✓	–	–
IPF#2	68	F	✓	–	–
IPF#3	63	F	✓	–	–
IPF#4	62	F	✓	–	–
IPF#5	50	M	✓	–	–
IPF#6	19	M	✓	–	–
IPF#7	62	M	✓	–	–
IPF#8	66	F	✓	–	–
IPF#9	59	M	✓	–	–
Do#1	74	F	✓	–	–
Do#2	57	M	✓	–	–
Do#3	14	F	✓	–	–
Do#4	54	M	✓	–	–
Do#5	32	M	✓	–	–
IPF#10	48	F	–	✓	–
IPF#11	38	M	–	✓	–
IPF#12	61	M	–	✓	–
IPF#13	56	M	–	✓	–
IPF#14	53	F	–	✓	–
IPF#15	35	F	–	✓	–
IPF#16	43	M	–	✓	–
IPF#17	57	M	–	✓	–
Do#6	31	F	–	✓	–
Do#7	58	F	–	✓	–
Do#8	37	W	–	✓	–
Do#9	72	W	–	✓	–
Do#10	60	M	–	✓	–
Do#11	54	F	–	✓	–
Do#12	53	F	–	✓	–
Do#13	60	M	–	✓	–
IPF#18	51	M	–	–	✓
IPF#19	50	F	–	–	✓
IPF#20	67	M	–	–	✓

### 2.2 Primary human lung fibroblasts

Primary human lung fibroblasts were isolated from explanted IPF (*n* = 8) and control lungs (*n* = 8) following an outgrowth technique as described before,^20^, ^21^ and all experiments were undertaken using cells from passage #3. Cells were grown in DMEM/F‐12 medium (with or without phenol red) supplemented with 10% FCS, 1% Penicillin/streptomycin, 1% nonessential amino acids and 1% L‐Glutamine. Lactate dehydrogenase (LDH) activity was measured by taking 50 µl of each supernatant according to the manufacturer's instructions (Sigma, #MAK066). Colorimetric bromodeoxyuridine (BrdU) assay (Sigma, #11647229001) was performed as described before^22^ by allowing cells to grow in a 96‐well plate followed by drug treatments as described below.

### 2.3 Precision cut lung slices

Low melting agarose (1.5–3%, maintained at 37°C) was filled in each segment of explanted human IPF lung and was allowed to cool on ice for 30 min for the agarose to solidify. Vibrating blade microtome (ThermoFisher Scientific) was used to section blocks of tissue filled with agarose. About 500‐µm thick sections were made and were cultured in RPMI medium without phenol red supplemented with 2% FCS, 1% penicillin/streptomycin and 1% L‐glutamine. PCLS were left for 24–48 h in cell culture incubator. PCLS were cultured in 6‐well plates, by pooling about three to five slices in one well, depending on the size of the PCLS used, and three such technical replicates were performed per each IPF lung used (three IPF patient lungs were used for the experiments undertaking PCLS samples, Table 1). Before treatments, PCLS were washed with PBS, and drug treatments were performed as described below.

### 2.4 Drug treatments and transfection

Pirf, and Ctd were dissolved in sterile‐filtered DMSO. Aza was dissolved in cold culture medium and was prepared freshly for each treatment. Hence, sterile‐filtered DMSO was used as vehicle‐treated control group (Veh). IPF fibroblasts or PCLS were treated for 24 h with pirfenidone (2.7 mM, Sigma, #P2116) as described before,^22^ Aza (2 µM, Sigma, #A2385) or Ctd (2.5 µM, 5 µM, Sigma, #C7632). Tandem fluorescence microscopy was performed using ptf‐LC3 (mRFP‐GFP‐LC3B; gift from Tamotsu Yoshimori, Addgene plasmid # 21074; http://n2t.net/addgene:21074; RRID: Addgene_21074). Cells were grown to 60% confluency and transfected with ptf‐LC3 plasmid using X‐tremeGENE HP DNA‐transfections reagent (Merck) as per manufacturer's instructions. Next day, drug treatments were performed as indicated for 24 h. Sterile‐filtered DMSO served as vehicle control. ‘*n*’ of 5–8 IPF patient fibroblasts were used and three technical replicates were performed for each treatment. Nontargeting control siRNA and BAG3 siRNA were obtained from Santa Cruz Biotechnology. siRNA transfections were performed using Lipofectamine 2000 following manufacturer's protocols and as described before^23^ for 48 h followed by treatments with the indicated drugs for additional 24 h.

### 2.5 Western blot and immunofluorescence

Denatured lung homogenates and cell lysates were used for western blotting following standard protocols as described before^20^, ^23^ to detect BAG3 (Proteintech, #10599‐1‐AP), BAG1 (Santa Cruz, #sc‐56005), p62 (Sigma Aldrich, #P0067), β‐actin (abcam, #ab8227), alpha SMA (abcam, #ab5694), FLNC (abcam, #ab180941), LC3B (Cell Signaling, #2775s), PCNA (Santa Cruz, #(PC10), sc‐56) and COL1A1 (Rockland, #600‐401‐103‐0.5). Autophagic flux was performed as described before^16^ following standard guidelines.^24^ Briefly, primary IPF fibroblasts were pretreated with 100 nM Bafilomycin A1 (Baf) or 30 µM Chloroquine (CQ) for 2 h followed by treatments with Aza or Ctd at the indicated concentrations for 4 h in the presence or absence of the inhibitors. Cells were then harvested to perform immunoblots for LC3B. Immunofluorescence was performed on primary fibroblasts and on formalin fixed, paraffin‐embedded PCLS sections of about 5 µm using standard protocols as described before^23^ and using antibodies against PCNA and COL1A1 as listed above. Microscopy was performed using a Leica M205 FA fluorescent stereoscope (Leica Microsystems) equipped with a Leica DFC360 FX camera. Image J was used to quantify about five to six regions from each group as described before^23^ and following the instructions in the Image J documentation: (https://www.unige.ch/medecine/bioimaging/files/1914/1208/6000/Quantification.pdf).

### 2.6 RNA isolation and qRT‐PCR

Total RNA was isolated using standard protocols as described before using RNeasy kit from Qiagen according to manufacturer's instructions and as described before.^25^ Concentration of RNA was measured using Nanodrop. Around 1 µg of RNA was used for cDNA synthesis using the PrimeScript RT Master Mix from Takara Bio Inc. Generated cDNA was diluted 1:10 using sterile RNase‐free water, and equal volumes were used as a template for amplification in a qRT‐PCR. Amplification was performed using the following primers for human BAG3: Forward primer: 5′TTCCACCAAGCCCAGAAGAC3′. Reverse primer: 5′CAGGTGCAGTTTCTCGATGG3’ and for the house keeping gene β‐actin using the following primers: Forward primer: 5′ACCCTGAAGTACCCCATCG3′. Reverse primer: 5′CAGCCTGGATAGCAACGTAC3’. Every reaction was performed as duplicates and quantified with the ΔΔC_T_‐method. Threshold cycles (C_T_) of target genes were normalized to a housekeeping gene (*ACTB*). The resulting Δ*C_T_* were compared to control samples (Veh), and relative mRNA expression was calculated by *R*  =  2^−ΔΔ^ *^C^_T_*.

### 2.7 Statistics

Data are expressed as means, ± SD. At least two independent experiments were performed from eight IPF and eight HD fibroblasts. About three to five PCLS from each IPF patient were pooled and PCLS from three IPF patients were used. The data did not follow normal distribution; hence statistical significance was assessed using nonparametric tests: Kruskal‐Wallis test followed by the Dunn's multiple comparison test for comparison between multiple groups and Mann–Whitney U test for comparing two groups. *p*‐value summary: *p≤0.05, **p≤0.01, ***p≤.001 or ^§^ *p*≤.5 or ^§§^ *p*≤.01, ^§§§^ *p*≤.001, ^§§§^ *p*≤.001.

## 3 RESULTS

### 3.1 Insufficient BAG3‐mediated autophagy and macroautophagy in IPF fibroblasts

**FIGURE 1:**
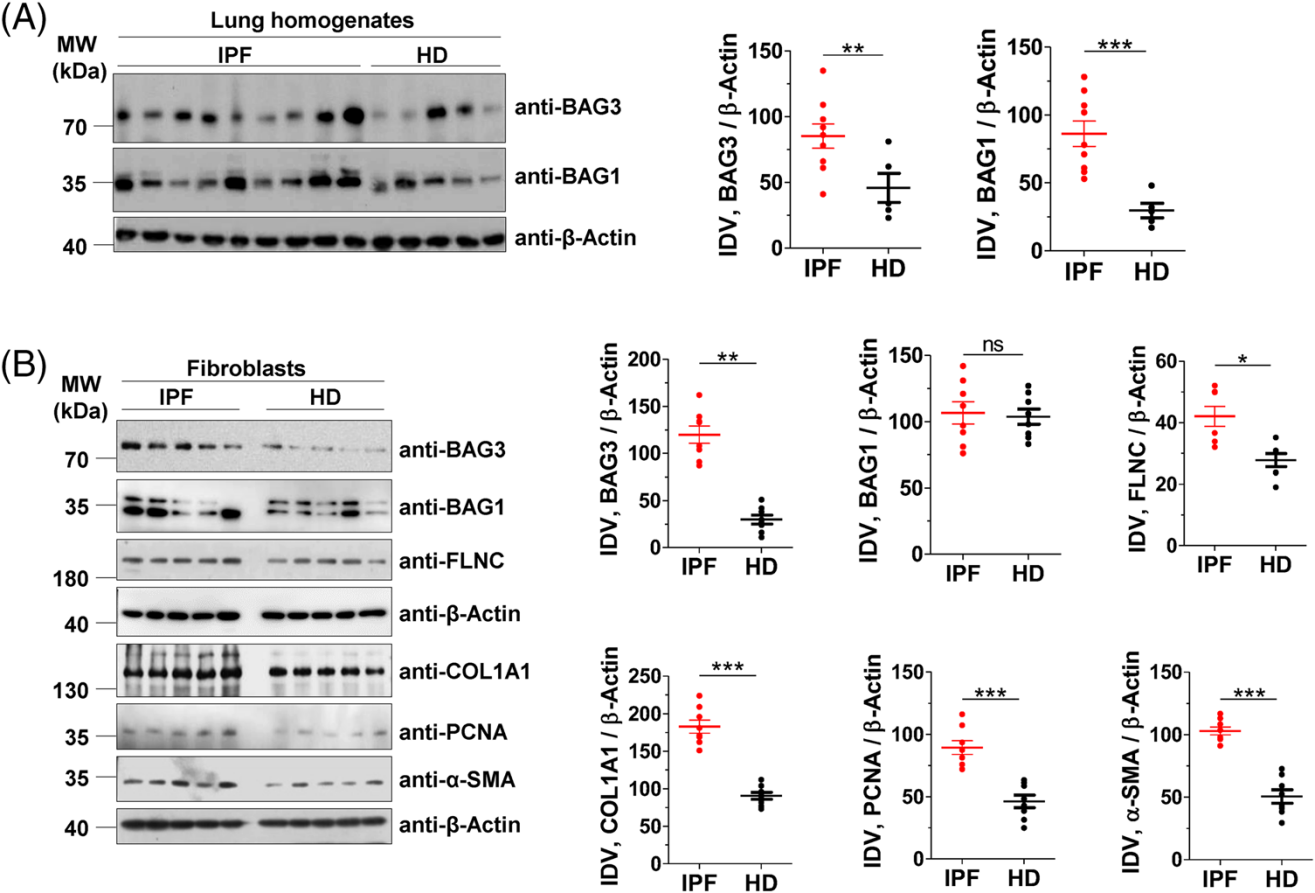
BAG3‐mediated autophagy is insufficient in IPF fibroblasts. (A) Immunoblot analysis of BAG3, BAG1 or β‐actin from lung homogenates (LH) of IPF patients or healthy donors (HD) (left). The right part shows respective quantifications after normalizing their integrated density values (IDV) from *n* = 9 IPF patients and 5 HD. (B) Immunoblots from cell lysates of primary interstitial fibroblasts of IPF or HD for the indicated proteins. The right part shows respective IDVs that were normalized to β‐actin from *n* = 8 each for IPF and HD. *p* value summary: *p ≤0 .05, ** *p* ≤ 0.01, *** *p* ≤0.001. *Abbreviation*: ns, not significant.

**FIGURE 2:**
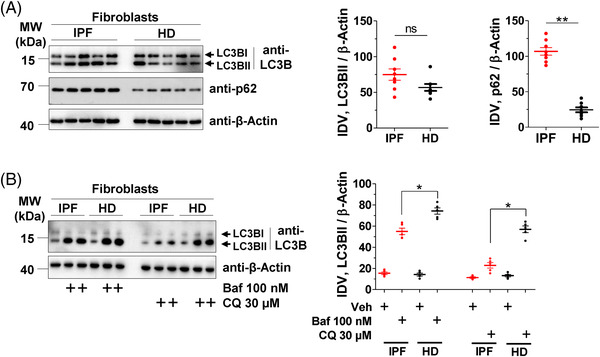
Insufficient autophagy in IPF fibroblasts. (A) Immunoblots from cell lysates of primary interstitial fibroblasts of IPF or HD for the indicated proteins. The right part shows IDVs of LC3BII or p62 were normalized to β‐actin. Blots and quantifications were performed from fibroblasts derived from *n* = 8 each for IPF and HD. (B) IPF or HD interstitial fibroblasts were pretreated with bafilomycin A1 (Baf) or chloroquine (CQ) for 2 h. Cells were lysed and the lysates were subjected to western blotting of LC3B and β‐actin. Two independent duplicates of treated IPF and HD samples were loaded along with one vehicle sample (left). IDVs for LC3BII were normalized to β‐actin (right) from *n* = 5 each for IPF or HD. *p*‐Value summary: * *p* ≤ 0.05, ** *p* ≤ 0.01, *** *p* ≤ 0.001. *Abbreviation*: ns, not significant.

While BAG3 is involved in the autophagy pathway, BAG1 has been reported to play important role in the ubiquitin‐proteasomal pathway.^1^, ^2^ In a first step, we compared BAG3 as well as BAG1 protein expression levels between IPF lungs and healthy donors (HD). The analysis of total lung homogenates (LH) of IPF patients revealed a significant increase in both BAG3 and BAG1 protein levels in IPF lungs as compared to those of HD (Figure 1A). Next, we analysed the expression of BAG3 in lysates from primary interstitial fibroblasts derived from IPF lungs or from HD. We found increased BAG3 but not BAG1 in IPF fibroblasts as compared to HD fibroblasts (Figure 1B). These observations prompted us to investigate the macroautophagy pathway, since BAG3 is majorly responsible for the recruitment of the macroautophagy pathway, and it has been shown that interstitial fibroblasts of IPF patients display defective autophagy.^15^ To test if BAG3‐mediated autophagy is altered in IPF fibroblasts, we analysed the expression of FLNC, a putative substrate of BAG3‐mediated autophagy. We observed a significant increase in FLNC protein levels in IPF fibroblasts as compared to HD (Figure 1B). These data indicate a defect in BAG3‐mediated selective autophagy in IPF fibroblasts. Alpha smooth muscle actin (α–SMA), COL1A1 and PCNA served as controls (Figure 1B). We also asked if defective autophagy is observed in IPF fibroblasts as reported by others. As shown in figure 2A, we did not observe a statistically significant difference in the autophagy marker protein, LC3BII, but we did observe a profound increase in the autophagy substrate protein, sequestosome 1 (SQSTM1/p62) in IPF fibroblasts as compared to those of HD. The increase in p62 protein may not fully reflect a decreased autophagic flux, as described in the guidelines of studying autophagy flux.^26^ We hence performed autophagic flux analysis where we treated IPF and HD fibroblasts with two different autophagy inhibitors, Baf and CQ. In the presence of these inhibitors, the increase in LC3BII protein levels in IPF fibroblasts were not as much as that observed in HD fibroblasts, indicating insufficient activation of autophagy‐related protein turnover in IPF fibroblasts (Figure 2B). This is in full accordance with the already published studies that showed a defective autophagy in IPF fibroblasts.^15^, ^27^

### 3.2 5 Aza and Ctd increase autophagy flux in IPF fibroblasts

**FIGURE 3:**
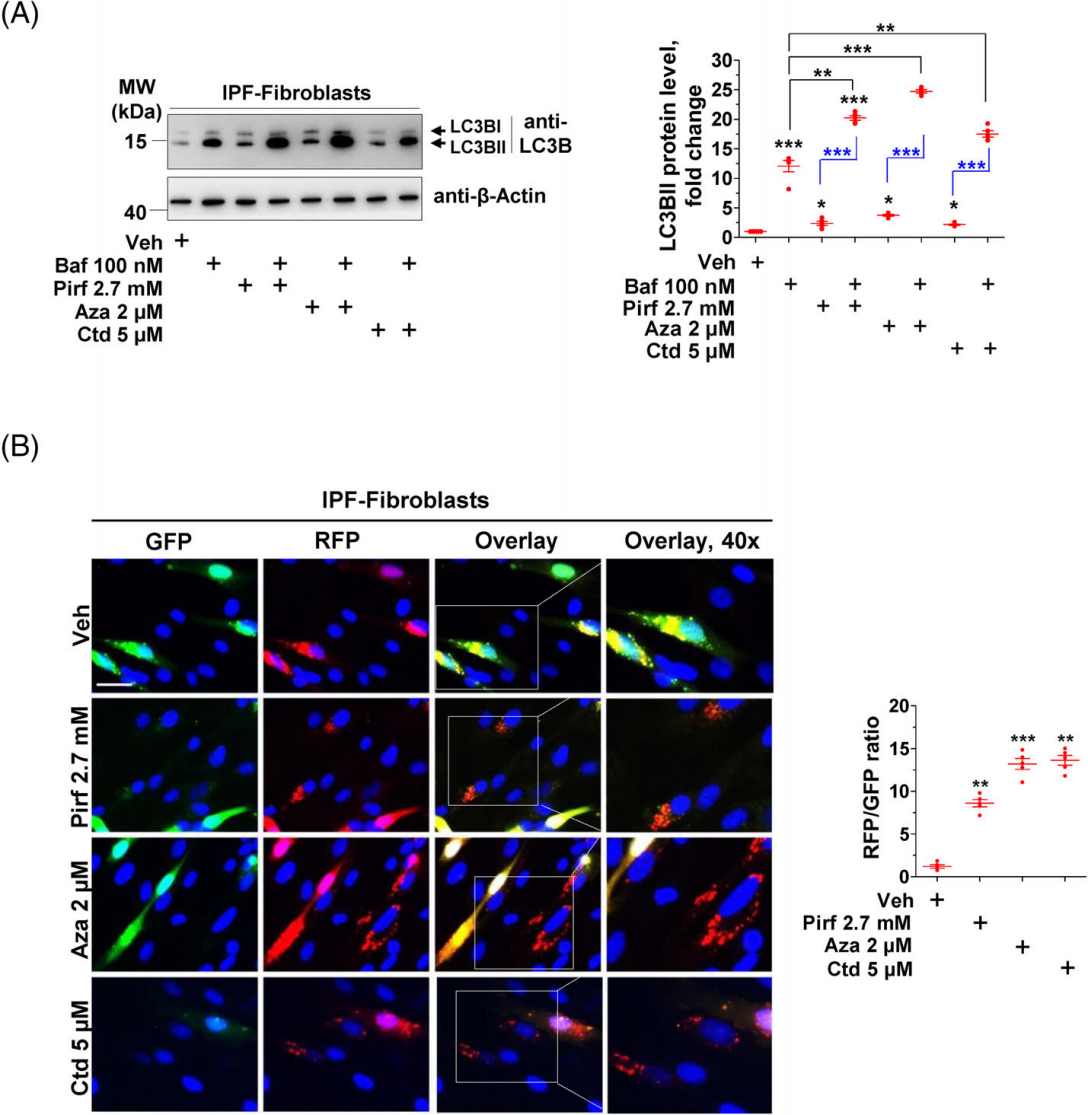
Pirfenidone (Pirf), 5‐azacytidine (Aza) and cantharidin (Ctd) increase autophagy flux in IPF fibroblasts. (A) IPF fibroblasts were pretreated with Baf, followed by treatments with vehicle (Veh), pirfenidone (Pirf), 5‐azacytidine (Aza), or cantharidin (Ctd) at the indicated concentrations for 4 h in the presence of Baf. Cells were lysed, and immunoblots were performed for LC3B and β‐actin. Relative LC3BII protein levels from *n* = 5 IPF patients were normalized to β‐actin. For the sake of understanding, LC3BII expression in Veh‐treated cell lysates was set as one. (B) IPF fibroblasts were transiently transfected with ptf‐LC3B (mRFP‐GFP‐LC3B) followed by treatments with Veh, Pirf, Aza or Ctd for 24 h at the indicated concentrations. Cells were then fixed and visualized by fluorescence microscopy for GFP or RFP puncta or for their co‐localization. Nuclei were stained with DAPI, scale bar = 15 µm. Puncta representing autophagosomes or autolysosomes or both were measured per cell using Image J, and their ratio was calculated for each treatment. Fibroblasts from five IPF patients were used for transfection, treatments and analysis. *p* Value summary: * *p* ≤ 0.05, ** *p* ≤ 0.01, *** *p* ≤ 0.001

In an attempt to investigate whether drugs interfering with autophagy may also affect the cell fate of IPF fibroblasts, we used two drugs: (1) Aza, a cytostatic drug with autophagy‐inducing properties, besides its well‐documented demethylation effects.^28^ Importantly, Aza was shown to inhibit the proliferation of IPF fibroblasts,^29^ but its effect on autophagy in these cells was not studied. (2) Ctd, a terpenoid that is primarily used as a phosphorylase inhibitor that is also known to inhibit BAG3 protein expression^30^ and to promote autophagy in the context of certain cancers.^31^ In a first step, we measured potential cytotoxic effects of Aza and Ctd in comparison to the established IPF drug Pirf. These experiments revealed nontoxic concentrations of Aza and Ctd (Figure S1), that were further used to test their effects on autophagy induction in IPF fibroblasts. We next treated IPF fibroblasts with Aza and Ctd in the presence or absence of the autophagy inhibitor, Baf in order to allow autophagy flux evaluation. Treatment of IPF fibroblasts with Aza, Ctd or Pirf resulted in a basal increase in the autophagy marker LC3BII as compared to vehicle‐treated cells and a further significant increase of LC3BII in the presence of Baf (Figure 3A). In addition, we transfected mRFP‐GFP tandem fluorescent‐tagged LC3 (ptfLC3) in IPF fibroblasts followed by treatments with vehicle (Veh), Pirf, Aza or Ctd to further study autophagy flux as described in the guidelines. Treatment of IPF fibroblasts with Pirf, Aza or Ctd, induced significantly increased red puncta, indicating the formation of autolysosomes, whereas in veh‐treated cells, yellow puncta are clearly visible representing accumulation of autophagosomes (Figure 3B). These readouts further strengthened our previous data, clearly indicating an increased autophagy flux by Aza, Ctd and Pirf in IPF fibroblasts.

### 3.3 Aza and Ctd modulate BAG3‐mediated autophagy and proliferation in IPF fibroblasts

**FIGURE 4:**
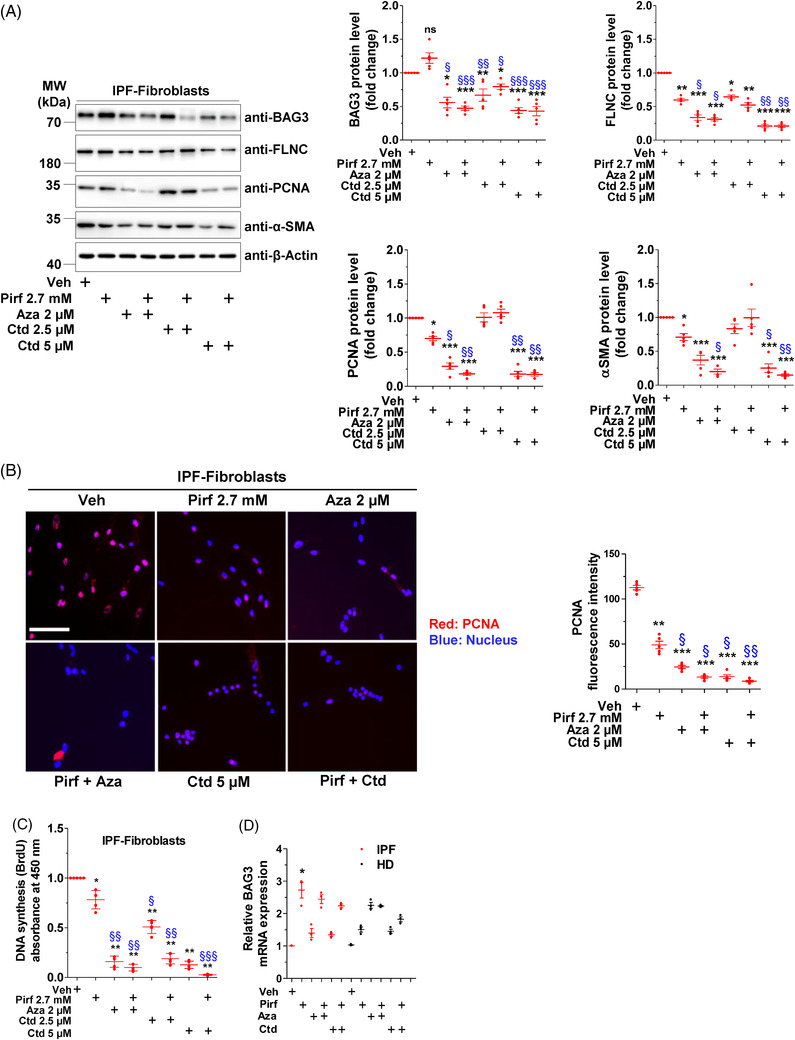
Pirf, Aza and Ctd activate BAG3‐mediated autophagy and decrease IPF fibroblast proliferation. (A) IPF interstitial fibroblasts were treated with Veh, Pirf, Aza or Ctd alone or Pirf in combination with Aza or Ctd as indicated for 24 h. Cells were lysed, and immunoblotting was performed for the indicated proteins (left). Relative protein amounts from five IPF patients were normalized to β‐actin and their fold change was obtained after the values in Veh‐treated controls was set as one (right). (B) Representative immunofluorescence staining for PCNA (red) in IPF fibroblasts after treating with Veh or the indicated drugs for 24 h. Nuclei were stained with DAPI (blue), scale bar = 25 µm. In the right panel, fluorescence intensity of PCNA was quantified using ImageJ. Analysis was performed from *n* = 5 IPF patient fibroblasts. (C) Proliferation of IPF fibroblasts (*n* = 5) as assessed by BrdU incorporation following indicated drug treatments for 24 h. Triplicates were measured, and absorbance values were set to one in Veh‐treated cells for the ease of understanding. (D) Analysis of BAG3 mRNA using qRT‐PCR in fibroblasts derived from IPF or HD lungs after treating them with the indicated drugs. Values were normalized to the house‐keeping gene β‐actin (*ACTB*). *BAG3* mRNA expression in Veh‐treated IPF/HD fibroblasts was set as one. *p*‐Value summary: * *p* ≤ 0.05, ** *p* ≤ 0.01, *** *p* ≤ 0.001 show significant difference as compared to Veh‐treated cells and ^§^ *p* ≤ 0.05, ^§§^ *p* ≤ 0.01, ^§§§^ *p* ≤ 0.001 indicate significant difference as compared to Pirf‐treated cells

We next analysed whether Aza, Ctd or Pirf would also influence the protein levels of BAG3 and its substrate FLNC. Pirf‐treated IPF fibroblasts did not reveal any changes in BAG3, but its substrate FLNC was significantly decreased. Treatment with either Aza alone or Ctd alone or in cells co‐treated with Prif–Aza or Pirf–Ctd, a significant decrease in BAG3 and its substrate FLNC was observed. In addition, the fibrosis marker α‐SMA as well as the proliferation marker, PCNA were also decreased (Figure 4A). Immunofluorescence analysis also showed a decreased staining for PCNA in response to the drug treatments, fully supporting the western blot data (Figure 4B). In addition, bromodeoxyuridine (5‐bromo‐2′‐deoxyuridine, BrdU) cell proliferation assay showed that the percentage of BrdU‐positive cells was significantly lowered by treatment with Pirf, Aza or Ctd alone as compared to vehicle‐treated cells. Interestingly, the combination of Pirf with Aza or Ctd led to an additional significant decrease of BrdU‐positive cells (Figure 4C). Of note, BAG3 mRNA did increase significantly in IPF fibroblasts treated with Pirf but did not significantly alter in response to Aza, Ctd or in combination with Pirf, although a trend towards increased BAG3 mRNA expression was observed (Figure 4D), indicating the action of these drugs on BAG3 protein level rather than its mRNA. In addition, like we did for IPF fibroblasts, we also treated primary donor lung fibroblasts, human embryonic kidney (HEK) cells and HeLa cells with Pirf, Aza, Ctd or with Pirf + Aza or Pirf + Ctd in order to study if these drugs exert similar effects on BAG3 protein levels in other cells. Interestingly, like in IPF fibroblasts, Aza and Ctd and their combination with Pirf decreased the protein levels of BAG3 these cell lines (Figure S2A–C).

### 3.4 Aza‐ and Ctd‐induced autophagy is BAG3 dependent

**FIGURE 5:**
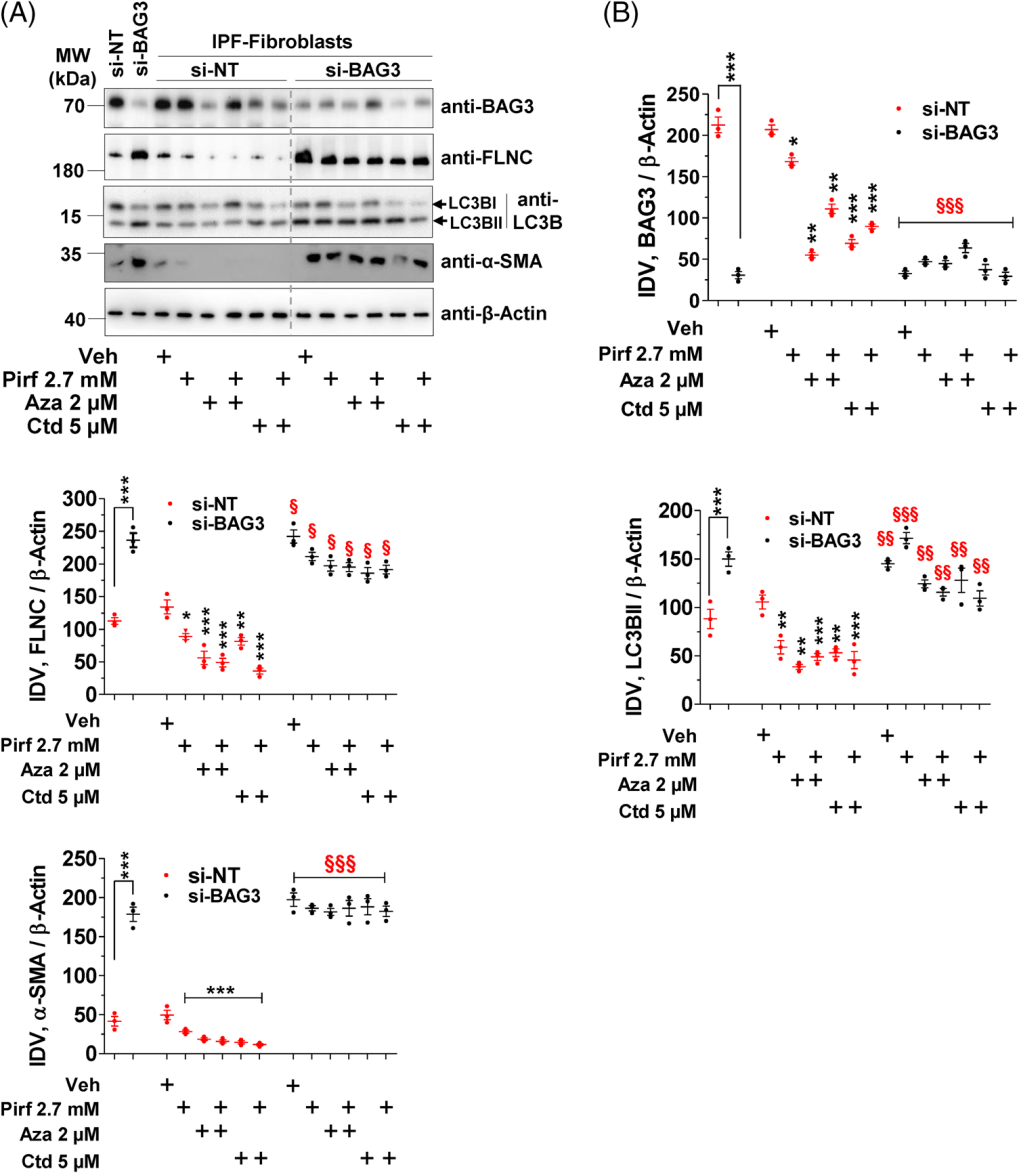
Therapeutic modulation but not siBAG3 per se affects autophagy in IPF fibroblasts. (A) IPF fibroblasts were transfected either with nontargeting siRNA (si‐NT) or with BAG3 siRNA (si‐BAG3) for 48 h followed by treatments with the indicated drugs for 24 h. Cell lysates were prepared, and western blots were performed for the indicated proteins. Bar graphs represent IDVs of BAG3, FLNC, LC3BII or α‐SMA that were normalized to β‐actin. Blots and analysis were performed from *n* = 3 IPF patient fibroblasts. *p* Value summary: *** *p* ≤ 0.001, significant difference between si‐NT and si‐BAG3 groups. * *p* ≤ 0.05, ** *p* ≤ 0.01, *** *p* ≤ 0.001 show significant difference between Veh‐treated and the respective drug treated groups in cells transfected with si‐NT. ^§^ *p* ≤ 0.05, ^§§^ *p* ≤ 0.01, ^§§§^ *p* ≤ 0.001 show significant difference between si‐BAG3 transfected cells with the indicated drug treatments versus their respective drug‐treated counterparts in si‐NT transfected cells

To further ascertain that these drugs impart their effects via BAG3 in IPF fibroblasts, we performed siRNA‐mediated knock down experiments for *BAG3* followed by drug treatments in these cells. As shown in Figure 5, we observed a significant decrease in BAG3 protein levels in IPF fibroblasts transfected with *siBAG3*. In IPF fibroblasts with *BAG3* knockdown, Pirf alone, Aza alone or Ctd alone or Pirf in combination with Aza or Ctd did not reduce the protein levels of FLNC as significantly as in the cells without *BAG3* knockdown in all treatment groups. Interestingly, the protein levels of LC3BII as well as the myofibroblast marker α‐SMA also did not decrease upon *BAG3* knockdown in any of the treatment groups (Figure 5). Collectively, these readouts indicated that the effects of Pirf, Aza and Ctd are at least in part mediated via activation of BAG3‐mediated autophagy.

### 3.5 Aza and Ctd decrease PCNA, BAG3 and collagen expression in precision cut lung slices of IPF patients

**FIGURE 6:**
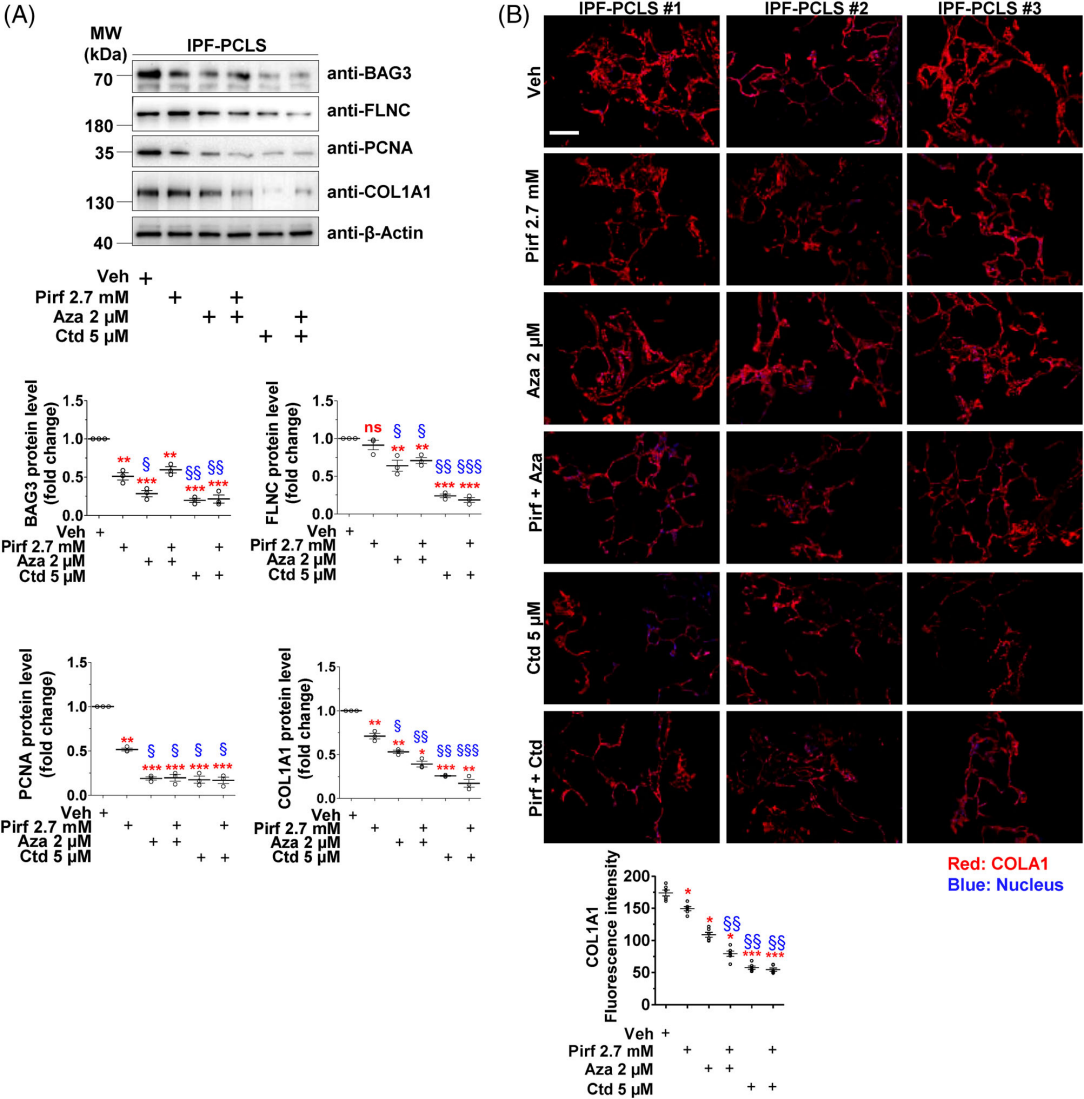
Therapeutic modulation of BAG3 decreases proliferation and COL1A1 in IPF PCLS. PCLS from explanted IPF patient lungs were treated with drugs, as indicated for 24 h. (A) PCLS lysates were prepared and tested for the indicated proteins via western blotting (top panel). Lower panels represent the quantifications of the indicated proteins after with β‐actin. Values in Veh‐treated PCLS were set as one. Treatment and representative analysis and blots from *n* = 3 IPF patients are shown. (B) Immunofluorescence staining for COL1A1 (red) on IPF PCLS upon Veh or drug treatments for 24 h has indicated. Nuclei were stained with DAPI (blue), scale bar = 200 µm. Lower panel indicates COL1A1 fluorescence intensity after drug treatments. Stainings and analysis were performed from *n* = 3 IPF PCLS per each group and about three technical replicates were performed. *p* Value summary: * *p* ≤ 0.05, ** *p* ≤ 0.01, *** *p* ≤ 0.001 show significant difference as compared to Veh‐treated PCLS and ^§^ *p* ≤ 0.05, ^§§^ *p* ≤ 0.01, ^§§§^ *p* ≤ 0.001 indicate significant difference as compared to Pirf‐treated PCLS

We next treated PCLS from explanted lungs of IPF patients with Pirf, Aza, Ctd alone or in combination. Western blot analysis revealed a significant decrease in the protein levels of the proliferation marker PCNA as well as the fibrosis marker collagen type I alpha 1 chain (COL1A1) in PCLS treated with Aza, Ctd or co‐treated with Pirf–Aza, Pirf–Ctd. These experiments also showed a decrease in protein levels of BAG3 and its substrate FLNC. Although Pirf treatment alone showed a significant decrease in BAG3, PCNA and COL1A1 protein levels, its effects were milder, especially as compared to Pirf+Aza or Pirf+Ctd groups. (Figure 6A). Further, immunofluorescence staining for extracellular COL1A1 revealed that, as compared to vehicle‐ or Pirf‐treated PCLS, Aza, Ctd, Pirf+Aza or Pirf+Ctd showed a significant decrease in COL1A1 staining (Figure 6B, S3).

## 4 DISCUSSION

**FIGURE 7:**
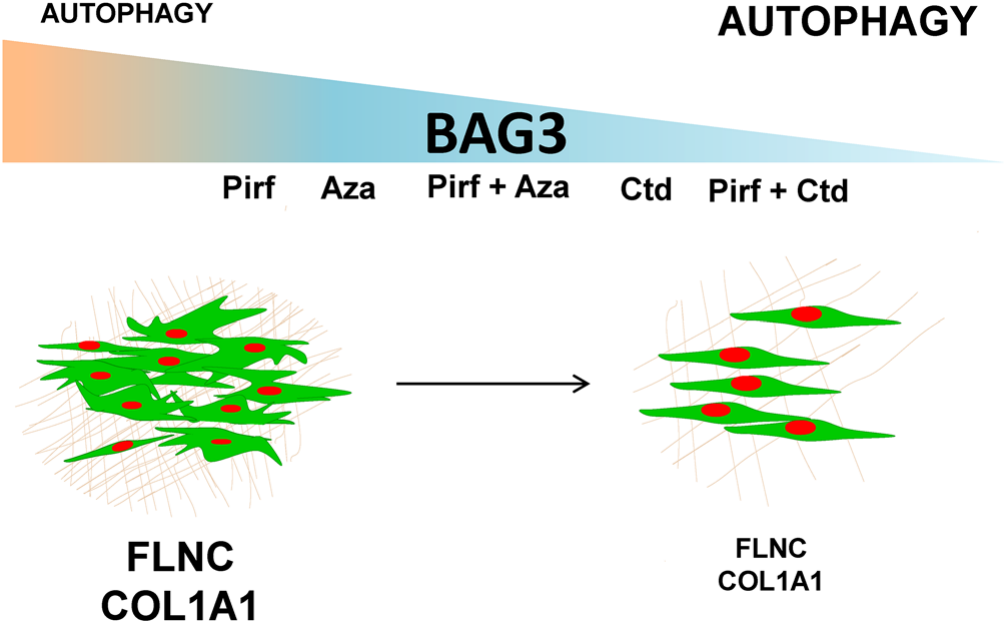
Schematic representation of this study. Restoring insufficient BAG3‐mediated autophagy in IPF fibroblasts by the indicated drugs. The slope represents the synergistic effect of the indicated drugs on BAG3 inhibition. Small font indicates decreased expression or decreased autophagy flux and vice versa. To the left, highly proliferative IPF fibroblasts (green) are depicted, enwrapped in thick extracellular matrix (brown) with increased BAG3, FLNC and COL1A1 and insufficient BAG3‐mediated autophagy. To the right, decreased IPF fibroblast proliferation, decreased FLNC, COL1A1 and BAG3 protein levels and restored autophagy upon treatments with the indicated drugs is shown

In this study, we identified insufficient BAG3‐dependent autophagy in fibroblasts of IPF patients. Administration of Aza and Ctd in both fibroblasts and in PCLS of IPF patients rescued BAG3‐dependent autophagy and decreased fibroblast proliferation alongside with a significant decrease in the fibrosis marker COL1A1 in PCLS from IPF patients (Figure 7). To our knowledge, this is the first study to reveal (a) the significant role of BAG3‐mediated autophagy and its proliferation‐regulating function in fibroblasts from IPF patients and (b) the BAG3‐mediated therapeutic efficacy of Aza and Ctd.

Reports in the past have revealed defective autophagy in both alveolar epithelial cells type II (AECII) and in fibroblasts of IPF patients.^15^, ^18^ In AECII, alongside with an accumulation of the autophagy substrate protein p62, PINK1‐dependent defective mitophagy was shown.^18^ In fibroblasts, accumulation of p62 and TGF‐β‐mediated defective autophagy were demonstrated.^15^ All these reports reveal that, although induction of autophagy is seen, both fibroblasts and AECII in IPF fail to perform competent autophagy. Supporting this, targeting the autophagy pathway using Rapamycin, a known inhibitor for mammalian target of rapamycin (mTOR) pathway, decreased the expression of fibrosis markers (alpha smooth muscle actin and fibronectin) in human fibroblast cell lines and lung hydroxyproline content in bleomycin model of lung fibrosis.^15^ Along this line, we also reported aberrant autophagy in two models of lung fibrosis, the Hermansky‐Pudlak syndrome (HPS)‐associated lung fibrosis as well as in the amiodarone model^16^, ^17^ and now, in primary lung fibroblasts of IPF patients. All these studies underscore the importance of classical macroautophagy and/or mitophagy in IPF and in animal models of lung fibrosis.

The molecular mechanisms responsible for the defective macroautophagy in IPF fibroblasts are not clear. Possible errors may occur in BAG3‐dependent selective degradation of cargo such as FLNC a process that occurs in an ubiquitin‐dependent manner, by closely cooperating with the HSP70‐associated E3 ubiquitin‐protein ligase CHIP.^32^ A further defective step during this process might be the efficient recruitment of p62 that facilitates cargo loading onto phagophore membranes.^9^

Defective autophagy has been implicated in several neurodegenerative diseases including Alzheimer's disease, Parkinson's disease and Huntington's disease.^33^ Autophagy activation using global mTORC inhibitors reduced neuronal toxicity in several diseases.^33^, ^34^ As the general relevance of autophagy prohibits its global therapeutic targeting, targeted modulation or fine tuning of autophagy were suggested as potential therapeutic strategies.^34^, ^35^ In this context, the three drugs that we used in this study, Pirf, Aza and Ctd are known to influence the autophagy pathway. Pirf is an approved drug for IPF therapy and affects several molecular mechanisms which ultimately lead to the inhibition of fibroblast proliferation.^22^ Interestingly, Pirf has been shown to promote mitophagy in lung fibroblasts.^36^ Aza is a well‐known DNA demethylating agent and a first‐choice therapy for myelodysplasia (MDS).^28^ It has been shown to increase autophagy and autophagic flux in bone marrow mononucleated cells of MDS patients after long‐term exposure to Aza.^28^ Clinically, it is a well‐tolerated drug, but rare cases of pneumonitis, interstitial lung disease and acute lung injury have been reported in individual reports but not in clinical trials.^37^, ^38^ One clinically noteworthy observation here is the co‐occurrence of MDS and IPF in several patients that could be ascribed to several risk factors including telomeropathies and aging,^39^ suggesting that overlapping mechanisms such as reduced autophagy in both diseases. In this study, Aza treatment resulted in increased autophagic flux, decreased fibrosis and reduced BAG3 and FLNC levels in IPF PCLS. Ctd reportedly inhibits autophagy in triple‐negative breast cancer cells,^40^ but in nonsmall cell lung cancer cells^31^ and human prostate cell lines,^41^ it increases autophagy flux. In addition, norcantharidin, an important derivative of Ctd, was shown to attenuate tubulointerstitial fibrosis^42^ and has been suggested to be a promising therapeutic target for renal fibrosis.^43^ Additionally, its distinct role in inhibiting BAG3 was shown in cancer cell lines.^30^ These BAG3 inhibiting, autophagy inducing and antifibrotic properties of Ctd were also clearly observed in our current study both in vitro and ex vivo. The combination of Ctd and Pirf showed additive effects potentially allowing the use of both substances at very low concentrations to avoid unwanted side effects.

A note of caution here is the apoptosis inducing nature of Aza and Ctd. It has been reported that Aza induces a dose‐dependent increase in apoptosis. In that, at concentrations between 2 and 6 µM, only cells at G1 phase preferentially underwent apoptosis.^44^ Another study revealed that Aza at 5 nM concentration neither affected the cell cycle nor increased apoptosis but sensitized lung cancer cells to cytotoxic drugs.^45^ It remains to be determined if the 2 µM dose used in our current study induces any apoptosis on fibroblasts or any other cell types on patient PCLS. Such dose‐dependent effects, in addition to time‐dependent apoptosis promoting features were also shown for Ctd in cancer cells.^46^ Although we did not observe severe apoptosis in our cells after 24 h of Ctd treatment at 5 µM dosage in IPF fibroblasts or in PCLS, in‐depth analysis are warranted to establish the extend of apoptosis not in fibroblasts but also in other types of cells. Another striking observation of our study is that Ctd treatment had hardly any effect on BAG3 mRNA. This is in contrast to an already published study that showed that Ctd in fact reduces the expression of BAG3 in human colorectal carcinoma cells.^30^ One straight forward explanation to this phenomenon could be cell type‐specific effects of Ctd; however, further studies are required to establish this.

It was suggested that BAG3‐mediated aggregation of critical cellular components that are required for an efficient autophagy‐lysosomal system may result in a defect in this pathway, providing a rational for the observation that decreased BAG3 leads to increased autophagy.^47^ In support to this notion in bronchial epithelial cells of cystic fibrosis (CF) patients, inhibition of BAG3 was shown to rescue defective autophagy. One study showed that MK2206, originally identified as AKT inhibitor, mechanistically targets BAG3 and rescues the autophagy pathway in ΔF508 CFBE41o‐ cells.^47^ A second study showed that *BAG3* silencing resulted in a functional correction of disease associated cystic fibrosis transmembrane conductance regulator (CFTR) variants including F508del‐CFTR.^48^ It will be interesting to see in future studies whether the autophagy‐inducing properties of Ctd, Aza and Pirf are restricted to fibroblasts as revealed here, or whether also other cell types such as AECII are affected.

## 5 CONCLUSIONS

To conclude, our study reveals that insufficient BAG3‐mediated autophagy is seen in IPF fibroblasts and may result their exaggerated proliferation. The BAG3 modulating drugs used here, namely Aza and Ctd, restored the autophagic flux to reduce fibroblast proliferation and collagen deposition and re‐established cellular homeostasis. These agents may thus be potentially usable in combination with clinically used Pirf and may represent a novel treatment approach in IPF by exerting further inhibitory effects on IPF fibroblast proliferation, altogether eliciting desirable therapeutic implications of targeted autophagy‐modulating drugs for IPF and maximize the efficacy of current IPF therapy.

## CONFLICT OF INTEREST

The authors declare no conflicts of interest.

## Supporting information



Supporting information

FigureS1

FigureS2

FigureS3

## Data Availability

The data that supports the findings of this study are available in the supplementary material of this article.
