# Supplementary material for: Therapeutic induction of Bcl2‐associated athanogene 3‐mediated autophagy in idiopathic pulmonary fibrosis
Source: Clin Transl Med. 2022 Jul 14;12(7):e935. doi: 10.1002/ctm2.935 (PMC9282656; doi:10.1002/ctm2.935)
Supplement: Supplementary file 1 — Supporting information [file CTM2-12-e935-s003.docx]

**Therapeutic induction of Bcl2-associated athanogene 3-mediated autophagy in idiopathic pulmonary fibrosis**

**Running title:** BAG3-mediated autophagy in IPF

Shashipavan Chillappagari^1,2,3^, Julian Schwarz^1^, Vidyasagar Kesireddy^1^, Jessica Knoell^1,2^, Martina Korfei^1,2^, Konrad Hoetzenecker^4,5^, M. Lienhard Schmitz^2,3,6^, Christian Behl^7^, Saverio Bellusci^1,2,6^, Andreas Guenther^1,2,5,6,8,^*^,#^, Poornima Mahavadi^1,2,^*^,#^

^1^Department of Internal Medicine, Justus-Liebig University (JLU) Giessen, Germany ^2^Universities of Giessen and Marburg Lung Center (UGMLC), Member of the German Centre for Lung Research (DZL), Giessen, Germany ^3^Department of Biochemistry, Faculty of Medicine, JLU Giessen, Germany ^4^Department of Thoracic Surgery, Vienna General Hospital, Vienna, Austria ^5^European IPF Network and European IPF Registry, ^6^Member of the Cardio-Pulmonary Institute (CPI), JLU Giessen, Germany ^7^Institute of Pathobiochemistry, The Autophagy Lab, University Medical Center, Johannes Gutenberg University, 55099 Mainz, Germany ^8^ Lung Clinic, Agaplesion Evangelisches Krankenhaus Mittelhessen, Giessen, Germany

*A.Guenther & P.Mahavadi contributed equally to this work

**^#^Corresponding authors:**

**Prof. Dr. Andreas Guenther**

Klinikstrasse 36, 35392 Giessen, Germany Tel. +49-641-985-42515

E-mail: [Andreas.Guenther@innere.med.uni-giessen.de](mailto:Andreas.Guenther@innere.med.uni-giessen.de)

**Dr. Poornima Mahavadi**

Gaffkystrasse 11, 35392 Giessen, Germany Tel. +49-641-99-42555

E-Mail: Poornima.Mahavadi@innere.med.uni-giessen.de

**Supplementary Information**

**Supplementary figure legends**

**Figure S1. Cytotoxity measurement in IPF fibroblasts.** IPF fibroblasts were treated with Pirf, Aza, or Ctd or with Pirf in combination with Aza or Ctd at different concentrations as indicated for 24 h. Supernatants were analyzed for LDH activity as described in methods. LDH activity in supernatants from Veh treated IPF fibroblasts was set as 100% and depicted here is LDH activity in different treatment groups relative to Veh treatments. *P≤0.05.

**Figure S2. Effects of Pirf, Aza and Ctd on BAG3 in different cell types. A.** Primary donor fibroblasts, **B.** HEK cells and **C.** HeLa cells were treated with the indicated drugs for 24 h and were subjected to western blotting of BAG3 and β-Actin. Graphs represent IDVs of BAG3 that were normalized to the respective β-Actins. At least two independent treatments were performed for each cell type. P value summary: *P ≤ 0.05, **P ≤ 0.01, ***P ≤ 0.001.

**Figure S3. Collagen stainings in IPF PCLS upon drug treatments.** 40x images of COL1A1 (red) immunofluorescence staining on IPF PCLS upon Veh or drug treatments as indicated for 24 h. Nuclei were stained with DAPI (blue). Scale bar = 100 μm.
