# Supplementary figures and images for: Therapeutic induction of Bcl2‐associated athanogene 3‐mediated autophagy in idiopathic pulmonary fibrosis
Source: Clin Transl Med. 2022 Jul 14;12(7):e935. doi: 10.1002/ctm2.935 (PMC9282656; doi:10.1002/ctm2.935)

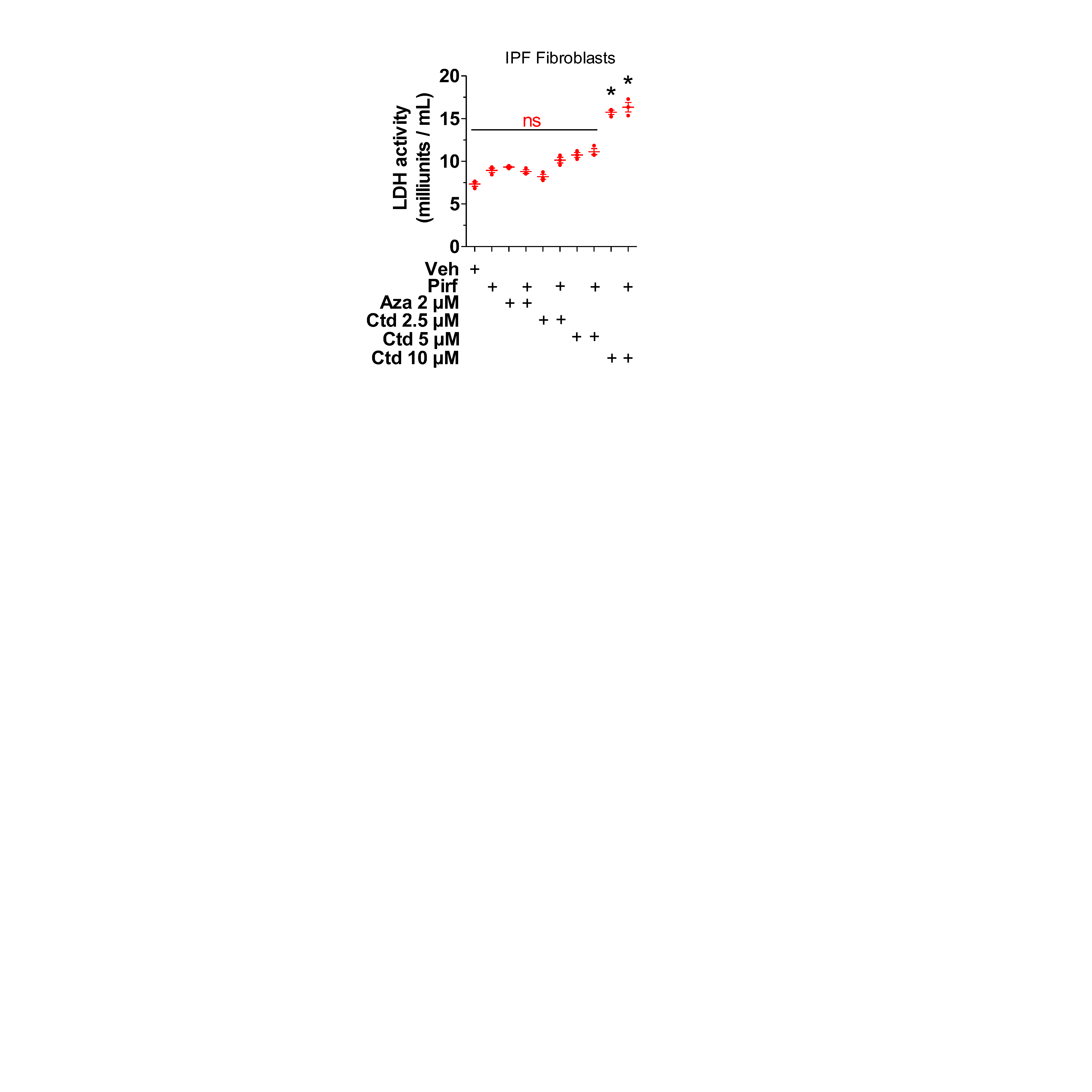

Supplement: Supplementary file 2 — FigureS1 [file CTM2-12-e935-s004.tiff]

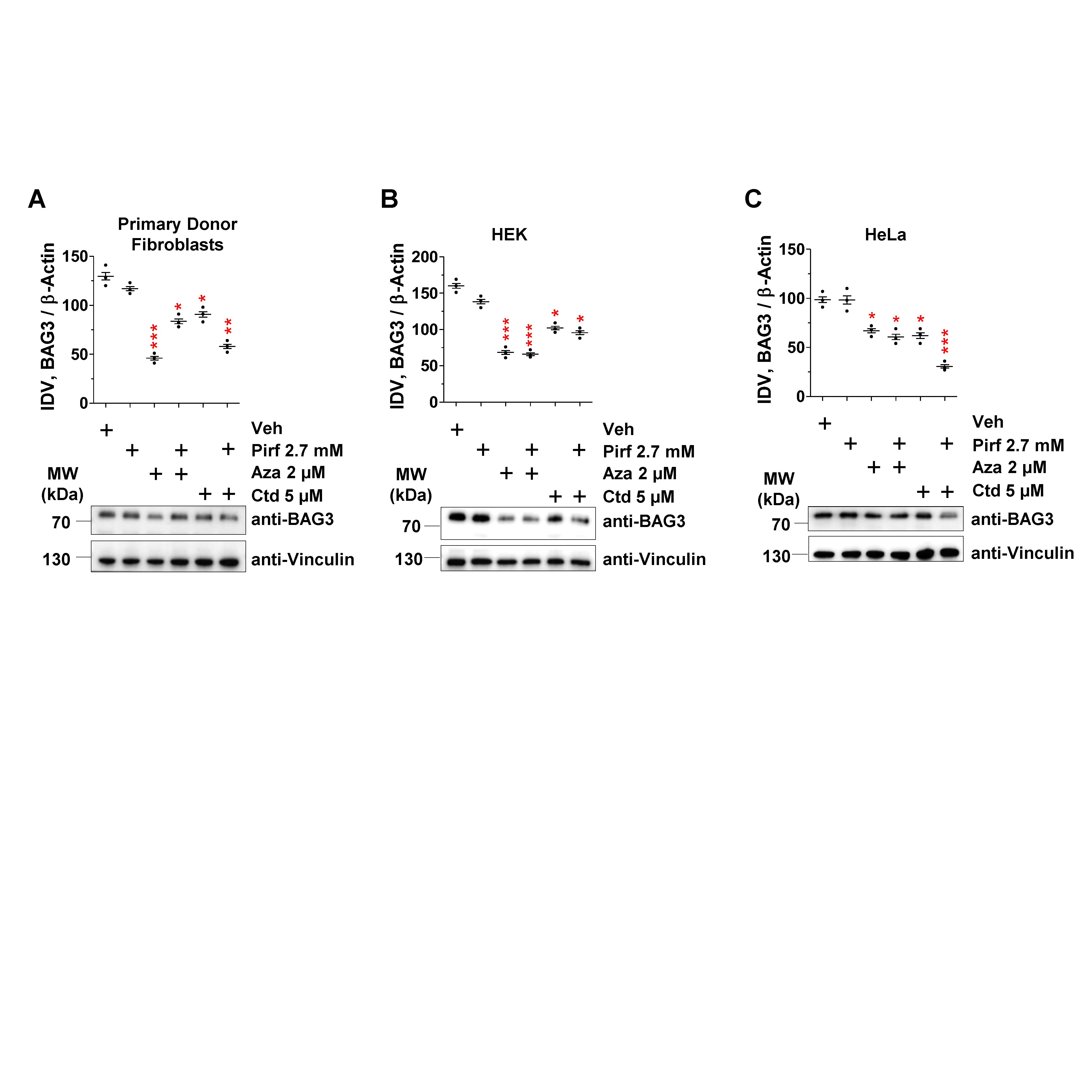

Supplement: Supplementary file 3 — FigureS2 [file CTM2-12-e935-s002.tiff]

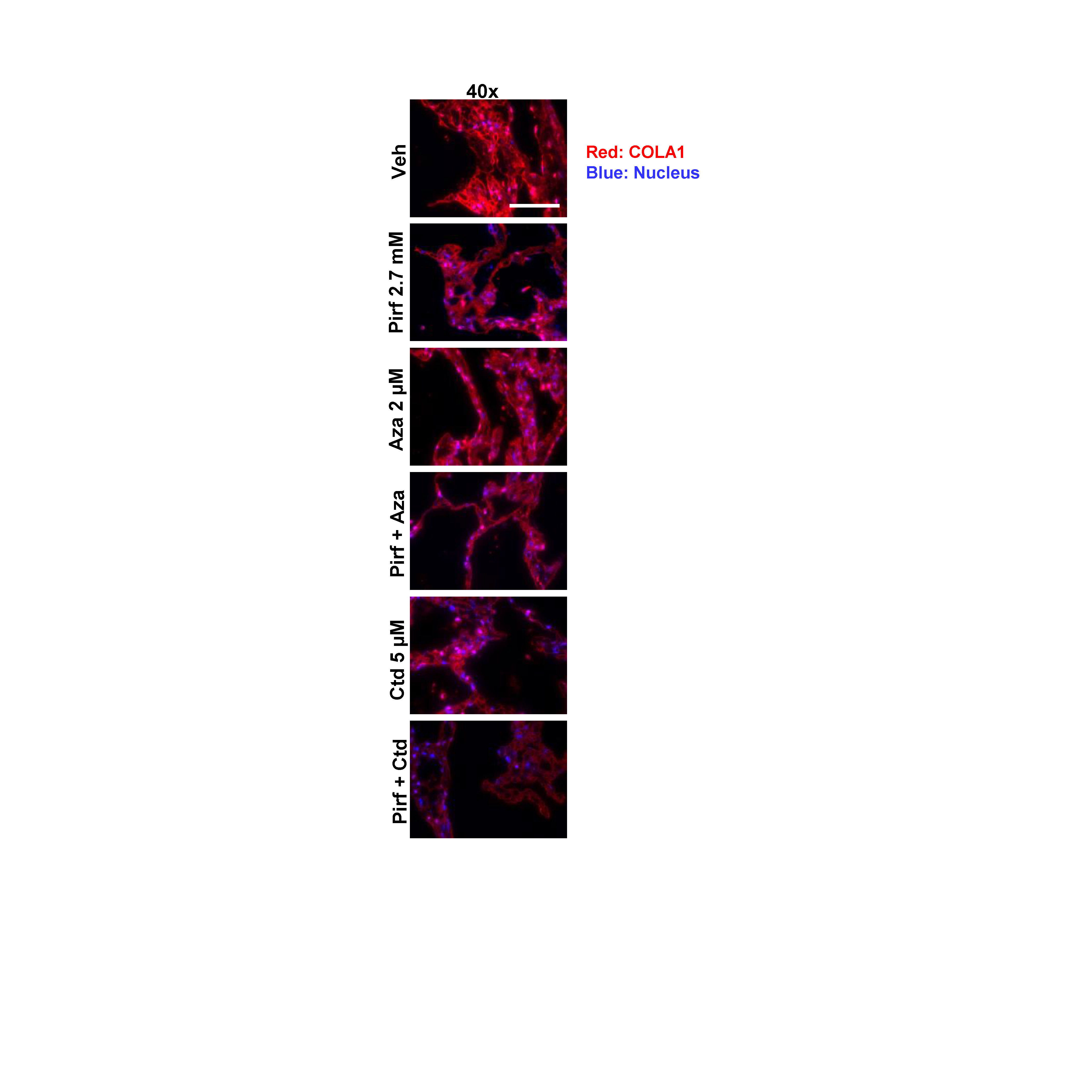

Supplement: Supplementary file 4 — FigureS3 [file CTM2-12-e935-s001.tiff]
